# Changes in Body Composition and Physical Performance in Wheelchair Basketball Players During a Competitive Season

**DOI:** 10.1515/hukin-2015-0102

**Published:** 2015-01-12

**Authors:** Aitor Iturricastillo, Cristina Granados, Javier Yanci

**Affiliations:** 1 Faculty of Physical Activity and Sports Science, University of the Basque Country, UPV/EHU, Vitoria-Gasteiz, Spain

**Keywords:** adapted sports, anthropometrics, skinfolds, physical performance, field tests

## Abstract

The present study analyzed the changes in body composition and physical performance in wheelchair basketball (WB) players during one competitive season. Players from a WB team competing in the first division of the Spanish League (n = 8, age: 26.5 ± 2.9 years, body mass: 79.8 ± 12.6 kg, sitting height: 91.4 ± 4.4 cm) participated in this research. The upper limbs showed a decrease in subcutaneous adipose tissue and there was an improvement in physical abilities such as sprinting with the ball (5 and 20 m), handgrip and aerobic capacity. However, the changes in physical fitness concerning sprinting without the ball and agility tests were low. It would be interesting to study the effects of implementing specific programs to improve physical performance in WB and to establish more test sessions to monitor the effects of the programs followed.

## Introduction

Wheelchair basketball (WB) is one of the most popular sports among the Paralympic disciplines and is practiced by people with different disabilities, according to the classification protocol of the International Wheelchair Basketball Federation (IWBF). WB is an intermittent sport which combines repeated high intensity sprints and rapid accelerations and decelerations with moderate and low intensity actions, with the purpose, among other aims, of achieving or maintaining a good position on the court (Molik et al., 2010). In this sense, both anaerobic and aerobic capacities are important for a better performance, during offensive and defensive situations (Molik et al., 2013). Body composition is also a significant factor affecting performance in WB.

Several studies have determined the importance of anthropometry in sport, and it is perhaps even greater in adapted sports, where certain players with spinal cord injury may experience a loss of metabolic activity in active fibers and muscle mass due to their particular injury (Collins et al., 2010). For this reason some authors have studied body composition using skin folds in wheelchair basketball and tennis players (Sutton et al., 2009). In this regard, some researchers have stated that both the player’s functional potential (Vanlandewijck et al., 2004) and their body composition (Goosey-Tolfrey et al., 2003) will influence physical performance in WB. Therefore, it could be important for coaches to study the evolution of the anthropometric characteristics of WB players during a competitive season.

Several articles have describe the physical qualities of WB players under laboratory conditions (Goosey-Tolfrey et al., 2003; Goosey-Tolfrey et al., 2008; Molik et al., 2010) and also using field tests carried out in their own playing area (De Groot et al., 2012; Vanlandewijck et al., 1999). Among the field tests, the most important are those that measure sprint capacity, change of direction ability and muscle strength (Goosey-Tolfrey et al., 2008; Molik et al., 2013; Yanci et al., 2015), and obviously those that measure aerobic capacity (Goosey-Tolfrey and Tolfrey, 2010; Vanlandewick et al., 1999). However, we have only found one study which monitored the changes in physical fitness of WB players (Ayán et al., 2014). Given the absence of research to corroborate these findings, more studies on this topic are needed to analyze the changes in physical performance during a competitive season.

Therefore, the purpose of the present study was to analyze the changes in body composition and physical performance in elite WB players during one competitive season.

## Material and Methods

### Participants

Players from a WB team competing in the first division of the Spanish League (n = 8, age: 26.5 ± 2.9 years, body mass: 79.8 ± 12.6 kg, sitting height: 91.4 ± 4.4 cm) participated in this research. All the subjects were informed about the risks and benefits of taking part in the study, knew that they could withdraw at any time, and signed a mandatory informed consent form.

### Procedures

The tests were carried out on the basketball court where the team trained. A week before the start of the official competitive season (pretest), different tests were performed to assess body composition and physical performance. At the end of the season, a week after the last league match (posttest) the same tests were repeated. On the first day the players performed the acceleration tests (5 and 20 m sprint with and without the ball) and the agility tests (T-test and Pick-Up test). On the second day (48 hours later) the measurements of body composition were taken, and the strength tests (maximal pass, medicine ball throw and handgrip) and the intermittent Yo-Yo Level 1 test of 10 m (YYIR1 10 m) were conducted. Before performing the test battery the players carried out a standard warm-up consisting of 5 min of low intensity wheelchair propulsion, two straight line accelerations of 10 m and two accelerations of 10 m with changes of direction. All the participants performed the tests in their usual wheelchair.

### Test battery

#### Body composition

The anthropometric variables of body mass (kg) and skin folds (mm) were measured in each player. Body mass was obtained to the nearest of 0.1 kg using an electronic scale (Seca^®^ Instruments Ltd., Hamburg, Germany) and four skin folds were measured (triceps, subscapular, suprailiac, and abdominal) with a skin fold caliper (Harpender Lange^®^, Cambridge, MA, USA) bearing in mind the indications specified by Goosey-Tolfrey et al. (2003). The same person recorded all the anthropometric variables on all occasions.

#### 5 and 20 m sprints (with and without the ball)

Three accelerations were performed over 5 and 20 m in a straight line with and without the ball (De Groot et al., 2012), with a 2 min rest period between each sprint, which was enough time to return to the start and wait for their next turn. The participants were placed at 0.5 m from the starting point and began when they felt ready. The timer was activated automatically as the subjects passed the first gate at the 0.0-m mark and split times were then recorded at 5 and 20 m (Granados et al., 2015). The time taken was recorded using three photocells (Microgate^®^ Polifemo, Bolzano, Italy). The best time was used for further analysis.

#### T-test

**Figure 1:**
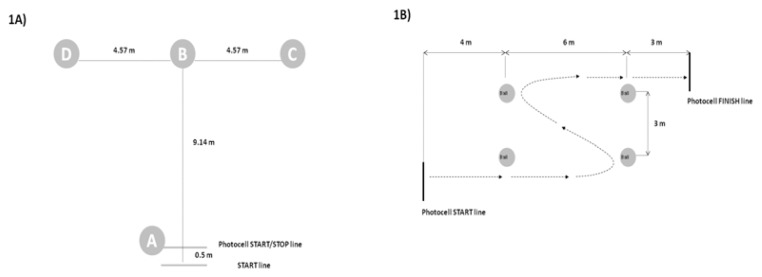
Agility T-test (1A) and Pick up test (1B)

The participants had to complete a circuit in the shape of a T according to the specifications of Yanci et al. (2015) for WB players. They began with the wheels 0.5 m from cone A, and completed the circuit as follows (Figure 1A), with modifications to allow performance in a wheelchair and always using forward movements (Yanci et al., 2015). Three repetitions were performed with 3 min rest between them. The time was recorded with a photocell (Microgate^®^ Polifemo, Bolzano, Italy). The best result was used for further analysis.

#### Pick-up

The test was performed three times with 3 min rest between, following the indications of De Groot et al. (2012). From a stationary position, the participant had to start propelling their chair and pick up 4 basketball balls from the floor twice with the left hand and twice with the right hand (Figure 1B). Three repetitions were performed with 3 min rest periods between them. Time was recorded using two photocells (Microgate^®^ Polifemo, Bolzano, Italy) placed at the beginning and the end of the course. The best time was used for further analysis.

#### Maximal pass

The subjects lined up at the marked line, with their front wheels behind the line, and performed three bilateral throws with a basketball trying to throw it as far as possible (De Groot et al., 2012). The distance was measured (m) between the marked line and the place where the ball bounced for the first time. The final score used for the statistical analysis was the average distance of five throws (De Groot et al., 2012).

#### Medicine ball throw

From the same position used in the *maximal pass* test, the players had to throw the 5 kg medicine ball as far as possible (Gonaus and Muller, 2012; Yanci et al., 2015). Each player was allowed three attempts and the distance was measured in m from the throwing line to the place where the ball made its first contact with the ground. The best of the three results was used for statistical analysis.

#### Handgrip

Forearm strength was measured in the dominant hand (Molik et al., 2013) using a portable hydraulic hand dynamometer (5030J1, Jamar^®^, Sammons Preston, Inc, United Kingdom). The test was performed in a sitting position in the wheelchair, with the arm in extension and in the vertical axis. The test protocol consisted of three maximal isometric contractions of 5 s, with a rest period of at least 60 s. The best of the three recordings was used for statistical analysis.

#### Yo-Yo 10 m Intermittent Recovery Endurance Test

Version 1 of the Yo-Yo test (YYIR1 10 m) was used as previously described by Yanci et al. (2015) and Granados et al. (2015) for WB players. The original YYIR1 test consisted of 20 m shuttle runs performed at increasing speed with 10 s of active recovery between runs until exhaustion. Considering the differences between running and propelling the wheelchair, the distance covered in the shuttle run was modified in this study to 10 m. The total distance covered (Castagna et al., 2008), a heart rate (HR) (Polar Team Sport System^®^, Polar Electro Oy, Finland) and lactate concentration (Lactate Pro LT-1710^®^, ArkRay Inc Ltd, Kyoto, Japan) were recorded. At the end of the endurance test, the subjects were also asked to rate their perceived exertion (RPE) on a 10-point category rating scale (Foster et al., 2001), presented on paper. They were asked separately for a respiratory rate of perceived exertion (RPEres) and an arm muscle rate of perceived exertion (RPEmus) as previously used in WB (Granados et al., 2015; Iturricastillo et al., in press).

### Training program and competition

During the competitive season the players competed in 16 official matches and trained twice per week. The training sessions consisted of an hour of exercise with and without the ball and the other hour was spent performing technical exercises and team tactics. The group tactics involved exercises with play situations in a more limited space and with fewer players. The training session always ended with real game situations.

### Statistical analysis

The statistical analysis was carried out with the Statistical Package for Social Sciences (SPSS^®^ Inc, version 20.0 Chicago, IL, USA.). The results are presented as mean ± standard deviation (SD). The normality of the data was analyzed with the Kolmogorov-Smirnov tests to verify the need for parametric or non-parametric tests. A t-test for related samples was used to determine the differences between the results obtained in the pre and posttest. The delta value (Δ%) between the pre and posttest was calculated using the formula: Δ% = [(Posttest-pretest)/pretest] × 100. The effect size (d) was calculated using the method proposed by Cohen (1988). Effect sizes lower than 0.2, between 0.2–0.5, between 0.5–0.8 or greater than 0.8 were considered trivial, low, moderate or high, respectively. Statistical significance was set at *p* < 0.05.

## Results

Table 1 shows the results of the body composition measurements in the pre and posttest. The players increased their body mass (p < 0.05, d = 0.30, low), and there was a tendency, although it was not significant, for a decrease in the triceps (p > 0.05, d = −1.40, high) and in the subscapular (p > 0.05, d = −0.50, moderate) skin fold, together with an increase in the suprailiac skin fold (p > 0.05, d = 0.55, moderate) at the end of the season.

With regard to physical performance, Table 2 shows the results in the pre and posttest of the players’ acceleration capacity with and without the ball and their agility. There was a tendency for improvement in acceleration over 5 m (p > 0.05, d = 0.79, moderate) and 20 m with the ball (p > 0.05, d = 0.44, low). However, no differences were found in acceleration without the ball or in agility (p > 0.05, range d = 0.11 – 0.33, low).

As to muscular strength, no significant differences were observed in the maximal pass distance between the pre and posttest (12.57 ± 2.10 m vs. 12.74 ± 2.41 m, p > 0.05, d = 0.08, trivial), or in the medicine ball throw (4.68 ± 0.54 m vs. 4.89 0.77 m, p > 0.05, d = 0.38, low). However, at the end of the season the players recorded better results in the handgrip test (p > 0.05, d = 1.26, high) than at the start of the season (Figure 1).

**Figure 2:**
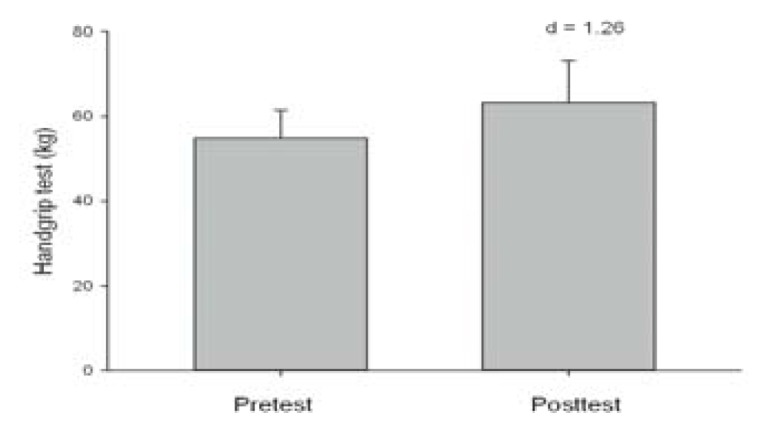
Mean values (± SD) obtained in the handgrip test in both the pre and posttest by WB players

With respect to the results obtained in the YYIR1, no differences were found between the pre and posttest in lactate concentration (9.33 ± 4.05 vs. 8.70 ± 3.56 mmol·l-1, p > 0.05, d = 0.16, trivial), or in the HRmax (184.5 ± 16.2 vs. 179.2 ± 14.1 latidos·min-1, p > 0.05, d = 0.33, low). However, at the end of the season, the players increased their performance with regard to total distance covered, although not significantly (p > 0.05, d = 0.77, moderate) (Figure 2). There was also a tendency for the respiratory RPE to be lower (6.58 ± 2.01 vs. 4.67 ± 3.14, p > 0.05, d = 0.95, high) but not the muscular RPE (5.92 ± 2.15 vs. 5.00 ± 2.53, p > 0.05, d = 0.43, low) in the post compared to the pretest.

## Discussion

The analysis of WB players’ physical fitness can provide relevant information for the determination of their sports performance (De Groot et al., 2012; Goosey-Tolfrey, 2005; Yanci et al., 2015), as it could help coaches improve the training process. The present study analyzed the evolution of body composition and physical performance in WB players during one competitive season. The results obtained regarding body composition revealed that there was an increase in body mass and a tendency for some skin folds to decrease. In relation to physical performance, the results in some physical fitness tests (5 and 20 m with the ball, distance covered in the YYIR1 10 m, and the handgrip test) were better at the end of the competitive season.

Several studies exist which have analyzed body composition in people with spinal cord injury (Collins et al., 2010; Price, 2010) and in female WB and wheelchair tennis players (Sutton et al., 2009). However, to our knowledge, there is no study that analyzes anthropometric characteristics of WB players during a competitive season. In our study, coinciding with Burke et al. (1986) who analyzed results before and after a competitive period in Australian rules football, there was a significant increase in body mass, but a decrease in skin folds. To be precise, the upper limbs showed a decrease in subcutaneous adipose tissue possibly due to a high level of activity when propelling the wheelchair, thus coinciding with the results obtained in the study by Sutton et al. (2009) where female WB players showed a lower percentage of adipose tissue in the arms than other players who did not use a wheelchair for sport. In this sense, continuous practice of WB aimed at reducing percentage of body fat could improve their physical performance and be beneficial for their health (Collins et al., 2010). In spite of the decrease in adipose tissue observed in some skin folds in the upper body (triceps and subscapular), the players in our study increased their suprailiac and abdominal skin folds at the end of the competitive season. It could possibly be interesting for WB coaches to control nutritional intake as body composition might have more likely been affected by diet rather than training and competition. A decrease in subcutaneous adipose tissue may be due to the training effects during the whole season.

Studies concerning changes in physical performance in WB during the competition period are scarce (Ayán et al., 2014) compared with able-bodied sports (Casajús, 2001; Drinkwater et al., 2005; Marques et al., 2006). The results obtained by these authors showed that physical performance in WB players revealed practically no changes during the season (trivial or low). In our case, the results were similar in both the pre and posttest (Δ% < 1.83%, d < 0.33, trivial or low) for the sprint tests without the ball, change of direction abilities and the throwing tests. However, contrary to the results presented by Ayán et al. (2014), the players in our study did obtain better results in the sprint tests of 5 and 20 m with the ball (Δ% = 7.84%, d = 0.79 and Δ% = 3,11%, d = 0.44, respectively) and in the handgrip test (Δ% = 19.78%, d = 1.26) at the end of the season, even though they did not follow any specific training during the season to improve their strength/power.

Possibly, and given that several studies on able-bodied sports had found a close association between strength and acceleration capacity (Granados et al., 2013; Rønnestad et al., 2011), and also in WB (Janssen et al., 1993; Molik et al., 2013; Turbanski and Schmidtbleicher, 2010), the absence of specific training aimed at improving strength could have influenced the lack of improvements in acceleration capacity, change of direction ability and throwing. It would therefore be interesting to analyze if including specific strength/power training sessions could produce improvements in the physical performance of WB players during the season.

Studies concerning changes in physical performance in WB during the competition period are scarce (Ayán et al., 2014) compared with able-bodied sports (Casajús, 2001; Drinkwater et al., 2005; Marques et al., 2006). The results obtained by these authors showed that physical performance in WB players revealed practically no changes during the season (trivial or low). In our case, the results were similar in both the pre and posttest (Δ% < 1.83%, d < 0.33, trivial or low) for the sprint tests without the ball, change of direction abilities and the throwing tests. However, contrary to the results presented by Ayán et al. (2014), the players in our study did obtain better results in the sprint tests of 5 and 20 m with the ball (Δ% = 7.84%, d = 0.79 and Δ% = 3,11%, d = 0.44, respectively) and in the handgrip test (Δ% = 19.78%, d = 1.26) at the end of the season, even though they did not follow any specific training during the season to improve their strength/power. Possibly, and given that several studies on able-bodied sports had found a close association between strength and acceleration capacity (Granados et al., 2013; Rønnestad et al., 2011), and also in WB (Janssen et al., 1993; Molik et al., 2013; Turbanski and Schmidtbleicher, 2010), the absence of specific training aimed at improving strength could have influenced the lack of improvements in acceleration capacity, change of direction ability and throwing. It would therefore be interesting to analyze if including specific strength/power training sessions could produce improvements in the physical performance of WB players during the season.

Aerobic capacity has also been studied in WB by different authors (Goosey-Tolfrey et al., 2008; Vanlandewick et al., 1999) due to its importance for the sport. In spite of the fact that field tests to measure changes in aerobic capacity have been widely applied in able-bodied team sports, only few papers have studied this quality in WB (Ayán al., 2015). Our results indicate that there was an improvement in the YYIR1 test at the end of the season, although it was not significant (Δ% = 14.73%, d = 0.77). Furthermore, the players who participated in our study showed lower levels of lactate concentration in the posttest (9.33 ± 4.05 mmol·l-1 vs. 8.70 ± 3.56 mmol·l-1). In this sense, WB players recorded a better performance in the YYIR1 and had better physiological responses at the end of the season; thus, the improvement in the YYIR1 test at the end of the season could indicate an improvement in aerobic capacity. There was also an improvement in their RPE, especially their RPEres (Δ% = 29.03%, d = 0.95), therefore, this fact confirms the better performance in the aerobic test at the end compared to the beginning of the season. These results are quite similar to those obtained by Ayán et al. (2012) as the latter state that they did not observe important changes in the multi stage fitness test (MSFT) at three different time points in the WB season (p = 0.683; Δ% = 10.40). Possibly the nature and typology of the tests used, the internal and external load in the training sessions and matches, as well as body composition at the respective moment could have influenced the obtained results. In this sense, it would be interesting to study the effects of implementing specific programs to improve physical performance in WB and to establish more test sessions to monitor the effects of the programs followed.

The obtained results should be interpreted with caution due to some important limitations of the study. First of all, nutritional intake during the whole season was not controlled, and the body composition changes might have been due to diet rather than training and competition. Moreover, a control group could have provided us with exact data on the training effects for physical fitness, which is why it would be interesting for future research to include one.

## Conclusions

In our study the WB players showed changes during the season in some variables of body composition and physical fitness, i.e. in acceleration capacity over 5 and 20 m with the ball, strength, the handgrip test, and the total distance covered in the endurance test. However, no differences were observed in acceleration capacity without the ball, change of direction ability, or explosive strength. Coaches of WB teams should consider the need to implement additional specific training sessions to improve these abilities in WB players.

## Figures and Tables

**Figure 3:**
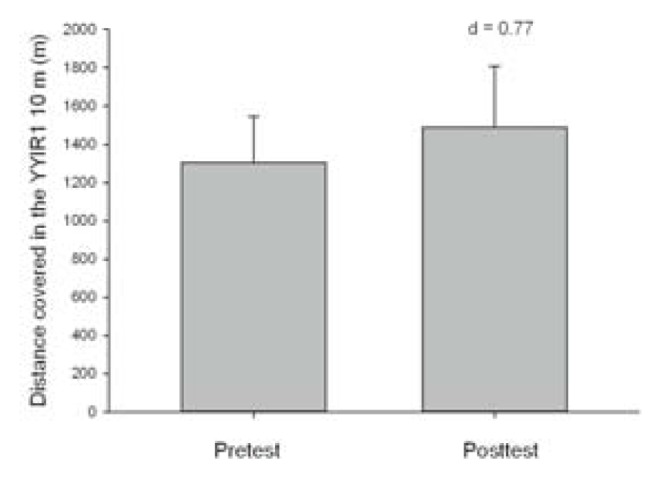
Distance covered in the Yo-Yo Level 1 10 m endurance test (YYIR1 10 m) in WB players

**Table 1:** Body composition in the pre and posttest in WB basketball players

	Pretest	SD	Posttest	SD	Δ(%)	d
Body mass (kg)	74.60	9.67	76.73^*^	10.11	2.86	0.30
Triceps	11.37	3.42	10.83	3.98	−4.77	−1.40
Subscapular	17.26	5.66	16.77	5.47	−2.81	−0.50
Suprailiac	15.60	6.71	16.17	6.85	3.66	0.55
Abdominal	28.94	11.49	30.20	10.04	4.34	0.38
Skin folds	69.08	24.97	64.73	33.59	−6.30	−0.25

SD = standard deviation, Δ = Difference of means, d = effect size

**Table 2:** Results of acceleration with and without the ball, and in the tests of change of direction ability both in the pre and posttest, in WB players

	Pretest (s)	Posttest (s)	Δ(%)	d
*Acceleration*				
Without B 5 m	1.73 ± 0.06	1.71 ± 0.10	−1.15	0.33
Without B 20 m	5.16 ± 0.18	5.18 ± 0.21	0.39	0.11
With B 5 m	1.89 ± 0.19	1.74 ± 0.15	−7.84	0.79
With B 20 m	5.76 ± 0.41	5.58 ± 0.38	−3.10	0.44
*Agility*				
T-Test	14.35 ± 0.62	14.14 ± 0.58	−1.42	0.33
Pick up	11.85 ± 0.78	11.64 ± 0.67	−1.83	0.28

Δ = difference of means, d = effect size, Without B = without ball, With B = with ball

## References

[b1-jhk-48-157] Ayán C, Cancela JM, Fernández B (2014). Changes in wheelchair basketball performance indicators throughout a regular season: a pilot study. Int J Perform Anal Sport.

[b2-jhk-48-157] Burke LM, Gollan RA, Read RS (1986). Seasonal changes in body composition in Australian Rules footballers. Br J Sports Med.

[b3-jhk-48-157] Casajús JA (2001). Seasonal variation in fitness variables in professional soccer players. J Sports Med Phys Fit.

[b4-jhk-48-157] Castagna C, Impellizzeri FM, Rampinini E, D’Ottavio S, Manzi V (2008). The Yo-Yo intermittent recovery test in basketball players. J Sci Med Sport.

[b5-jhk-48-157] Cohen J (1988). Statistical power analysis for the behavioral sciences.

[b6-jhk-48-157] Collins EG, Gater D, Kiratli J, Butler J, Hanson K, Langbein WE (2010). Energy cost of physical activities in persons with spinal cord injury. Med Sci Sports Exerc.

[b7-jhk-48-157] Croft L, Dybrus S, Lenton J, Goosey-Tolfrey VL (2010). A comparison of the physiological demands of wheelchair basketball and wheelchair tennis. Int J Sports Phys Perform.

[b8-jhk-48-157] De Groot S, Balvers IJ, Kouwenhoven SM, Janssen TW (2012). Validity and reliability of tests determining performance-related components of wheelchair basketball. J Sports Sci.

[b9-jhk-48-157] Drinkwater E, Hopkins WG, McKenna MJ, Hunt PH, Pyne DB (2005). Characterizing changes in fitness of basketball players within and between seasons. Int J Perform Anal Sport.

[b10-jhk-48-157] Foster C, Florhaug JA, Franklin J, Gottschall L, Hrovatin LA, Parker S, Doleshal P, Dodge C (2001). A new approach to monitoring exercise training. J Strength Cond Res.

[b11-jhk-48-157] Gonaus C, Müller E (2012). Using physiological data to predict future career progression in 14- to 17-year-old Austrian soccer academy players. J Sports Sci.

[b12-jhk-48-157] Goosey-Tolfrey VL, Batterham AM, Tolfrey K (2003). Scaling behavior of VO_2peak_ in trained wheelchair athletes. Med Sci Sports Exerc.

[b13-jhk-48-157] Goosey-Tolfrey VL (2005). Physiological profiles of elite wheelchair basketball players in preparation for the 2000 Paralympic games. Adapt Phys Activ Q.

[b14-jhk-48-157] Goosey-Tolfrey VL, Foden E, Perret C, Degens H (2008). Effects of inspiratory muscle training on respiratory function and repetitive sprint performance in wheelchair basketball players. Br J Sports Med.

[b15-jhk-48-157] Goosey-Tolfrey V, Tolfrey K (2010). The multi-stage fitness test as a predictor of endurance fitness in wheelchair athletes. J Sports Sci.

[b16-jhk-48-157] Granados C, Izquierdo M, Ibañez J, Ruesta M, Gorostiaga EM (2013). Are there any differences in physical fitness and throwing velocity between national and international elite female handball players?. J Strength Cond Res.

[b17-jhk-48-157] Granados C, Yanci J, Badiola A, Iturricastillo A, Otero M, Olasagasti J, Bidaurrazaga I, Gil SM (2015). Anthropometry and Performance in Wheelchair Basketball. J Strength Cond Res.

[b18-jhk-48-157] Iturricastillo A, Yanci J, Granados C, Goosey-Tolfrey VL Quantifying wheelchair basketball match load: a comparison of heart rate and perceived exertion methods. Int J Sports Phys Perform.

[b19-jhk-48-157] Janssen TW, van Oers CA, Hollander AP, Veeger HP, van der Woude LH (1993). Isometric strength, sprint power and aerobic power in individuals with a spinal cord injury. Med Sci Sports Exec.

[b20-jhk-48-157] Marques MC, van den Tilaar R, Vescovi JD, Gonzalez-Badillo JJ (2007). Relationship between throwing velocity, muscle power, and bar velocity during bench press in elite handball players. Int J Sports Phys Perform.

[b21-jhk-48-157] Molik B, Laskin JJ, Kosmol A, Skucas K, Bida U (2010). Relationship between functional classification levels and anaerobic performance of wheelchair basketball athletes. Res Q Exerc Sport.

[b22-jhk-48-157] Molik B, Laskin J, Kosmol A, Marszalek J, Morgule-Adamowicz N, Frick T (2013). Relationships between anaerobic performance, field tests, and level of elite female wheelchair basketball athletes. Hum Mov Sci.

[b23-jhk-48-157] Price M (2010). Energy Expenditure and Metabolism during Exercise in Persons with a Spinal Cord Injury. Sports Med.

[b24-jhk-48-157] Rønnestad B, Nymark BS, Raastad T (2011). Effects of in-season strength maintenance training frequency in professional soccer players. J Strength Cond Res.

[b25-jhk-48-157] Sutton L, Wallace J, Goosey-Tolfrey VL, Scott M, Reilly T (2009). Body composition of female wheelchair athletes. Int J Sports Med.

[b26-jhk-48-157] Turbanski S, Schmidtbleicher D (2010). Effects of Heavy Resistance Training on Strength and Power in Upper Extremities in Wheelchair Athletes. J Strength Cond Res.

[b27-jhk-48-157] Vanlandewijck YC, Daly DJ, Theisen DM (1999). Field test evaluation of aerobic, anaerobic, and wheelchair basketball skill performances. Int J Sports Med.

[b28-jhk-48-157] Vanlandewijck YC, Evaggelinou C, Daly DJ, Verellen J, Van Houtte S, Aspeslagh V, Hendrickx R, Piessens T, Zwakhoven B (2004). The relationship between functional potential and field performance in elite female wheelchair basketball players. J Sports Sci.

[b29-jhk-48-157] Yanci J, Granados C, Otero M, Badiola A, Olasagasti J, Bidaurrazaga I, Iturricastillo A, Gil SM (2015). Sprint, agility, strength and endurance capacity in wheelchair basketball players. Biol Sport.

